# Fruit Architecture in Polyamine-Rich Tomato Germplasm Is Determined via a Medley of Cell Cycle, Cell Expansion, and Fruit Shape Genes

**DOI:** 10.3390/plants8100387

**Published:** 2019-09-29

**Authors:** Raheel Anwar, Shazia Fatima, Autar K. Mattoo, Avtar K. Handa

**Affiliations:** 1 Department of Horticulture and Landscape Architecture, 625 Agriculture Mall Drive, Purdue University, West Lafayette, IN 47906, USA; raheelanwar@uaf.edu.pk (R.A.); shaziafatima66@gmail.com (S.F.); 2 Institute of Horticultural Sciences, University of Agriculture, Faisalabad, Punjab 38040, Pakistan; 3 Sustainable Agricultural Systems Laboratory, U.S. Department of Agriculture, Agricultural Research Service, The Henry A. Wallace Beltsville Agricultural Research Center, Beltsville, MD 20705, USA

**Keywords:** putrescine, spermidine, spermine, tomato, spermidine synthase, fruit shape, cell division, cell expansion

## Abstract

Shape and size are important features of fruits. Studies using tomatoes expressing *yeast Spermidine Synthase* under either a constitutive or a fruit-ripening promoter showed obovoid fruit phenotype compared to spherical fruit in controls, suggesting that polyamines (PAs) have a role in fruit shape. The obovoid fruit pericarp exhibited decreased cell layers and pericarp thickness compared to wild-type fruit. Transgenic floral buds and ovaries accumulated higher levels of free PAs, with the bound form of PAs being predominant. Transcripts of the fruit shape genes, *SUN1* and *OVATE*, and those of *CDKB2*, *CYCB2*, *KRP1* and *WEE1* genes increased significantly in the transgenic ovaries 2 and 5 days after pollination (DAP). The levels of cell expansion genes *CCS52A*/*B* increased at 10 and 20 DAP in the transgenic fruits and exhibited negative correlation with free or bound forms of PAs. In addition, the cell layers and pericarp thickness of the transgenic fruits were inversely associated with free or bound PAs in 10 and 20 DAP transgenic ovaries. Collectively, these results provide evidence for a linkage between PA homeostasis and expression patterns of fruit shape, cell division, and cell expansion genes during early fruit development, and suggest role(s) of PAs in tomato fruit architecture.

## 1. Introduction

Domestication of tomato has led to different phenotypes, including diversity in fruit shape, color, and size [1]. QTL mapping and genomic analyses have identified several loci underlying the observed diversity in shape and size of tomato fruit [1,2,3,4,5]. Genes that regulate fruit architecture include shape and size genes, namely, *CNR/FW2.2* [6], *OVATE* [2], *FAS* [7], *SUN1* [8], *fw11.3* [9], *LC* [10,11], *fs8.1* [12], and *KLUH*/*FW3.2* [13]. The *FW2.2* locus encodes a protein with homology to a cell-membrane-localized ras-like G-protein, which has been implicated in controlling ~47% of variation in fruit mass in *Solanum pimpinellifolium* and *S. pennellii* [6,14]. A mutation in the *FW2.2* promoter inhibits cell division during flower development and causes a larger fruit phenotype [15]. Another gene *SUN1* encodes a protein harboring an IQ67 domain and affects cell number along the entire proximal-distal axis, resulting in fruit elongation [16,17,18]. *OVATE* family proteins (OFPs) and TONNEAU1 Recruiting Motif proteins affect fruit shape by regulating cell division patterns during ovary development [19,20]. It is known that a mutation in the carboxyl-terminal domain of *OVATE* results in changing fruit shape from round- to pear-shaped [2]. *SUN* has a stronger effect on transcriptome than *OVATE* and *fs8.1*. Auxin has been implicated in regulating the expression of some of the fruit shape genes [18], but little is known about other molecules that may affect fruit size and shape.

Polyamines (PAs)—putrescine (Put), spermidine (Spd), and spermine (Spm), are ubiquitous polycations in all organisms, including plants, implicated in a myriad of developmental and physiological processes, including cell proliferation [21,22,23,24]. In plants, PAs play roles in biotic and abiotic stresses [25]; fate of flower, fruit, and seed development [26]; leaf and flower senescence [27,28]; in vitro somatic embryogenesis and organogenesis [29,30]; and fruit ripening and shelf life [27,31]. PAs homeostasis is a genetically regulated process with tight PAs homeostasis in most organisms and their perturbation results in altered phenotypes [26]. Exogenous application of PAs was found to increase the expression of cell division genes *CYCA* and *CYCB* in tobacco BY-2 cell cultures [32]. Overexpression of *KRP1*, a cyclin-dependent kinase inhibitor 1, affected cell division and led to several altered phenotypes in plants, including flower morphology and plant size [33].

PAs are predominantly present during growth and development in plants, in particular during cell division and elongation in apical shoots and meristems before flowering [21,34,35]. A crosstalk among various PAs and plant hormones has been implicated in several growth and developmental processes [36]. Transgenic tomato fruits expressing *CDKA1* under the control of a fruit-specific promoter increased cell division, resulting in higher thickness of pericarp and placenta, and larger septa and columella [37]. We previously developed isogenic tomato lines homozygous for expression of yeast Spd synthase (*ySpdSyn*) with altered levels of Spd and Spm in developing and ripening tomato fruit [27]. These isogenic transgenic fruits with altered PA homeostasis resulted in architectural changes in fruit such as a more obovoid phenotype, compared to an otherwise spherical phenotype in wild-type (WT) fruit. The altered phenotype of transgenic fruit manifests during early fruit development and is associated with higher levels of Spd/Spm. Our results show strong positive correlations between transcript levels of fruit shape-regulating genes (*SUN1* and *OVATE*), cell cycle genes (*CDKB2*, *CYCB2*, *KRP1* and *CCS52B*), and endogenous levels of free and bound PAs. These findings provide first molecular evidence for the role of PAs as fruit architecture regulators responsible for the obovoid phenotype of tomato fruit.

## 2. Results

### 2.1. Transgenic Expression of ySpdSyn Caused Architectural Changes in Tomato Fruit

**Figure 1:**
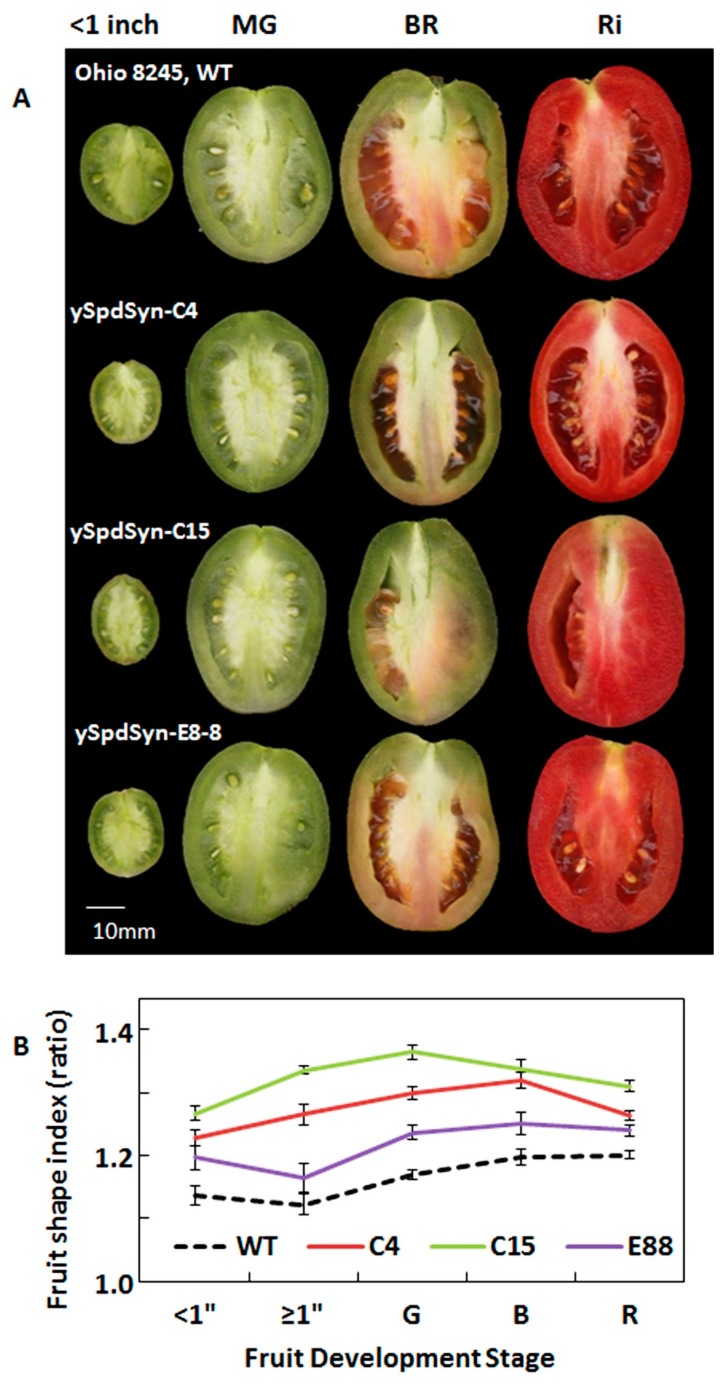
Morphometric changes in transgenic tomato fruit expressing *ySpdSyn*. Phenotype (**a**) and fruit shape index (**b**) of field grown wild-type (WT, wild-type cv. Ohio 8245) and transgenic fruits expressing *ySpdSyn* under CaMV35S (C4 and C15) and fruit specific E8 (E8-8) promoters. The fruits shown represent average growth and development stage of indicated genotype. The white line in the bottom left corner (Figure 1a) represents 10 mm on the original scale. Vertically cut tomato fruits were scanned and analyzed with tomato analyzer 3.0 software (Figure 1b). Fruit shape index is length-to-width ratio of the fruit. Error bars in Figure 1b represent standard error of means (n > 3 biological replicates, where each replicate had at least 50 tomato fruits). Abbreviations: MG or G—mature green stage; BR or B—breaker stage; Ri or R—red ripe stage of tomato fruit development.

The isogenic tomato germplasm homozygous for transgene *ySpdSyn* under the control of either constitutive CaMV35S (lines C4 and C15) or fruit-specific SlE8 promoters (line E8-8) have been previously described [27]. Fruits from the *ySpdSyn* transgenic lines exhibited an obovoid shape, compared to the relatively spherical phenotype of parental Wild-type cv. Ohio 8245 fruits (Figure 1a,b). The altered phenotype transition—from more spherical to more obovoid—of the transgenic fruit occurs at early stages of fruit development and continues thereafter to full maturity (Figure 1a,b). The effect of transgene was more pronounced under the constitutive CaMV35S promoter than under the developmentally regulated fruit-ripening SlE8 promoter. Collectively, these results associate the expression of *ySpdSyn* transgene with the observed altered tomato fruit architecture from round to obovoid.

**Figure 2:**
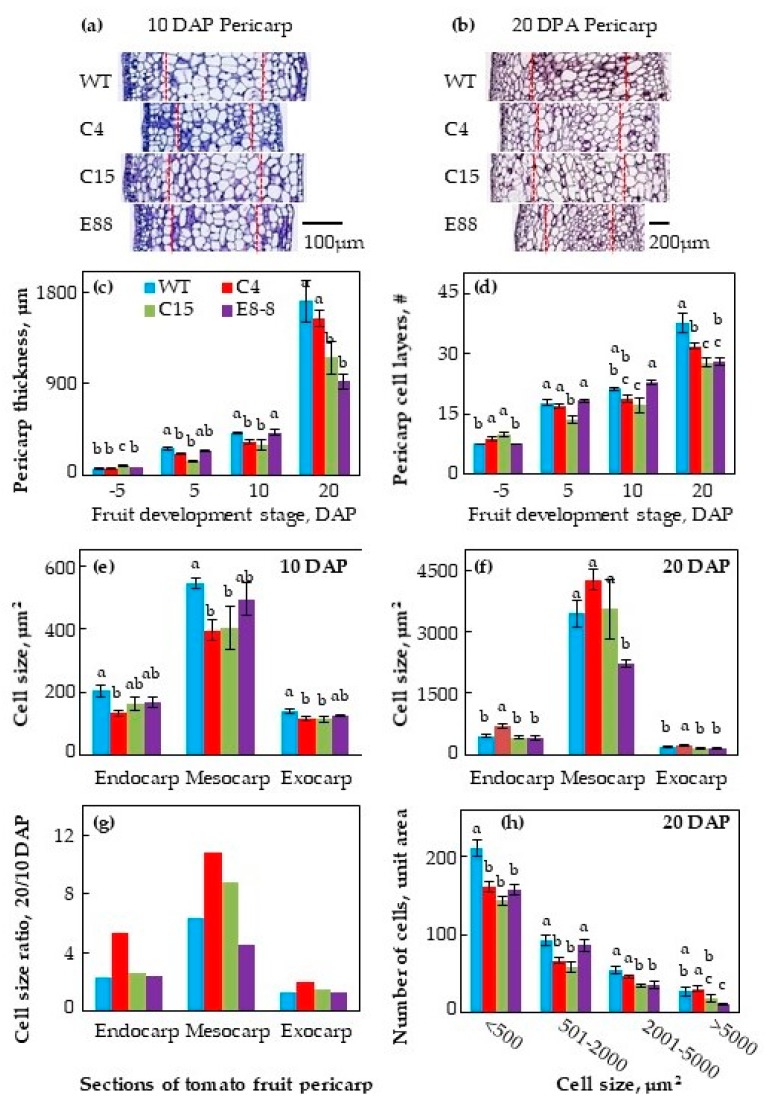
Histological analysis of WT and transgenic fruitlets at 5 days before pollination and at 5, 10 and 20 days after pollination (DAP). Toluidine blue–O staining of WT and transgenic fruitlets at (**a**) 10 DAP and (**b**) 20 DAP. (**c**) Changes in pericarp thickness; (**d**) number of anticlinal cell layers in pericarp; (**e**) cell size at 10 DAP and (**f**) 20 DAP; (**g**) cell size ratio of 20 DAP/10 DAP in endocarp (single innermost cell layer), mesocarp (middle 50% of the pericarp), and exocarp (2 outer cell layers) of tomato ovaries; (h) number of cells in each category of cell area within each genotype. Flowers were tagged and ovaries from flowers at 5 days before pollination and at 5, 10, and 20 DAP were fixed in 100% methanol, vertically sectioned and stained with 0.04% toluidine blue–O. Digital images of pericarp sections were acquired using AperioScan and analyzed using ImageScope 11. Average cell size (**e**,**f**) was calculated by dividing the total number of cells with the area of endocarp, mesocarp, or exocarp. Shown are average ± standard error (n ≥ 3 biological replicates). Different letters above the standard error bars indicate significant difference (at 95% confidence interval) among genotypes within the pericarp section.

The cytological examination of pericarp cell layers, cell size, and cell thickness of developing ovaries of WT and the three transgenic lines was performed at 5 days before pollination (DBP) and 5, 10 and 20 days after pollination (DAP) to ascertain the nature of the structural alterations. The medial-lateral sections of the fruit pericarp tissue from WT and transgenic lines at 10 and 20 DAP were stained with 0.04% toluidine blue–O, and are shown in Figure 2a,b; quantified data for the pericarp thickness and cell layer number from −5 to 20 DAP are shown in Figure 2c,d, respectively. The thickness of pericarp in all the three independent transgenic lines, particularly C15 and E8-8 fruit, was significantly reduced compared to WT fruit (Figure 2c), which was associated with a decreased number of cell layers at 20 DAP (Figure 2d). On an average, fruit pericarp of WT fruit tissues at 20 DAP had 38 cell layers while the transgenic fruit pericarp from C4, C15, and E8-8 lines had 32, 28, and 28 cell layers, respectively (Figure 2d). The cell sizes in endocarp, mesocarp, and exocarp of the transgenic fruit were reduced compared to WT fruits, with significant reduction in the cell size of the 10 DAP mesocarp of C4 and C15 lines, as compared to WT fruit mesocarp (Figure 2e). At 20 DAP, however, the mesocarp cell size was unchanged in fruit of C4 and C15 lines, but significantly decreased in E8-8 fruit compared to WT fruit (Figure 2f). The cell sizes in 20 DAP fruit increased by 2- to 11-fold in endocarp, mesocarp, and exocarp, compared to the 10 DAP fruit, implicating a shift from cell division to cell expansion mode (Figure 2g). This increase was much larger in endocarp and mesocarp of the transgenic C4 and C15 fruits compared to WT fruit at 20 DAP.

In order to determine the phenotypic basis of reduced pericarp thickness of the fruit from transgenic lines, we evaluated the distribution of smaller and larger cells in mediolateral pericarp slices. The pericarp of the transgenic fruits from C4, C15, and E8-8 lines had a reduced number of cells per unit area than the WT pericarp (Figure 2h). Reduction in cell size was seen in almost all cell types, small to large. However, the percent of total distribution of small and large cells ranging from ≤500 to ≥5000 remained similar in all the genotypes (data not shown).

### 2.2. Expression of ySpdSyn Transgene and SlSpdSyn in Floral Buds and Fertilized Ovaries

**Figure 3:**
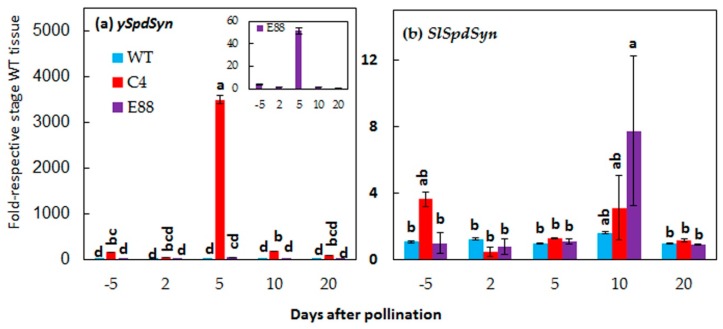
Changes in steady state levels of (**a**) *ySpdSyn* and (**b**) *SlSpdSyn* transcripts during early development of WT and *ySpdSyn* expressing tomato fruits. Total RNA from three biological replicates of floral buds at 5 days before pollination, and flower ovaries at 2, 5, 10, and 20 days after pollination were independently extracted and reverse transcribed. The levels of *ySpdSyn* and *SlSpdSyn* transcripts were determined using qRT-PCR with gene-specific primers (Table S3). The inset in upper panel shows the transcript levels of *E8:ySpdSyn* transgene in E8-8 tissues at a higher magnification. The relative expression levels were calculated by the 2^−ΔΔCт^ method using *SlACTIN* (Solyc04g011500.2.1) as housekeeping gene and plotted as fold-respective to WT tissues. Shown are average ± standard error. Statistical analyses were performed using the XLSTAT ANOVA method using −5 DAP WT as reference.

The expression of *ySpdSyn* in floral buds and fertilized ovaries of the transgenic and WT tomato lines was quantified by qRT-PCR to evaluate the association between transgene expression and altered phenotype. The *CaMV35S:ySpdSyn* transgene was expressed in C4 tissues as early as 5 DBP and in the fertilized ovaries at 2 to 20 DAP. The transgene expression in C4 was much higher in 5 DAP ovaries than the other stages of floral buds and ovaries examined (Figure 3a). Transcripts of *E8:ySpdSyn* transgene were also detectable at 5 DBP and 5 DAP stages, but the levels were much lower than the *CaMV35S:ySpdSyn* transcripts at all the fruit developmental stages examined (Figure 3a inset). Expression of the endogenous *SlSpdSyn* gene was also regulated during the floral bud development, with high levels observed in 5 DBP and 10 DAP fruits (Figure 3b). Other stages had low levels of *SlSpdSyn* transcripts (Figure 3b). Transgene under the CaMV35 promoter increased *SlSpdSyn* transcripts at 5 DBP and that under SlE8 at 10 DAP stages of fruit development (Figure 3b). Constitutive expression of *ySpdSyn* in C15 line exhibited upregulated expression of endogenous *SlSpdSyn* gene to as much as 5.8-fold in 10 DAP ovaries when compared with WT fruit tissues (Table S1).

### 2.3. Expression Patterns of Fruit Size and Shape Genes in Transgenic Fruits

**Figure 4:**
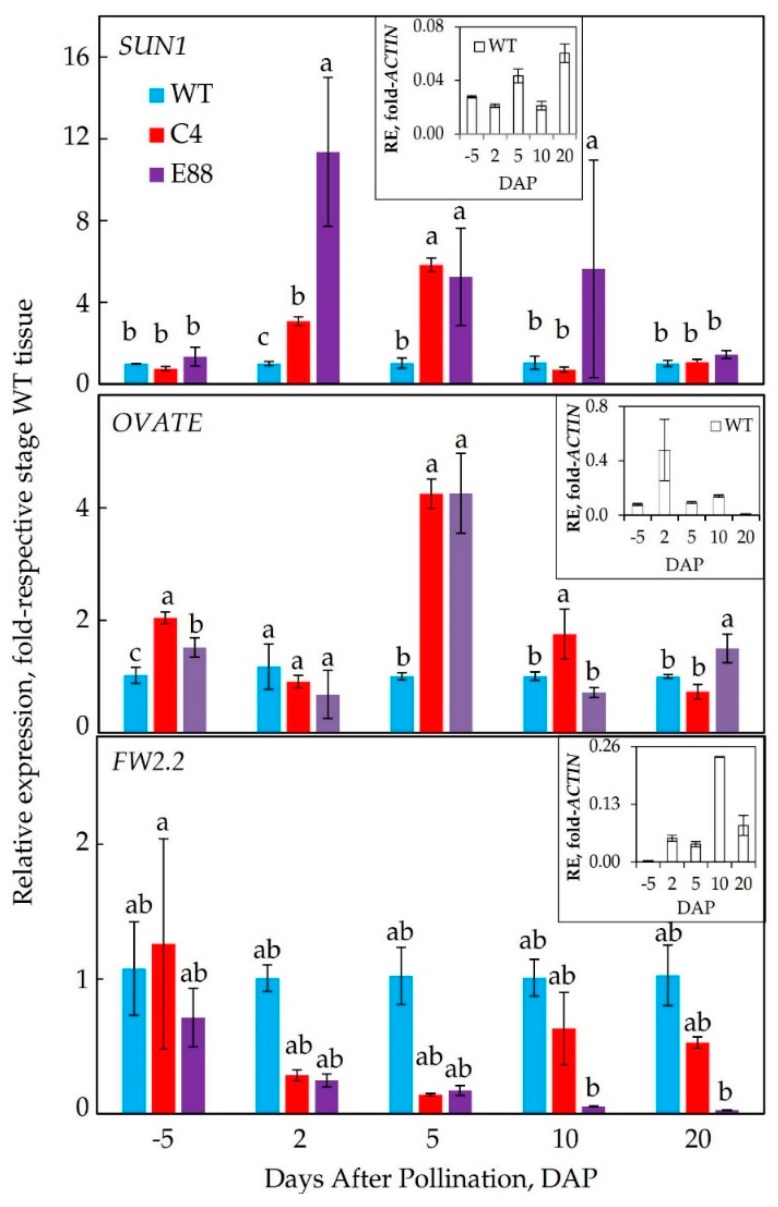
Changes in transcript levels of fruit shape-related genes *SUN1*, *OVATE*, and *FW2.2* in WT and *ySpdSyn*-expressing transgenic tomato floral buds and flower ovaries. Transcripts were quantified using qRT-PCR and relative expression levels were calculated by the 2^−ΔΔCт^ method using *SlACTIN* (Solyc04g011500.2.1) as housekeeping gene and plotted as fold-respective to WT tissues. Other details are the same as in the legend in Figure 3 legend.

The expression patterns of *SUN1*, *OVATE*, and *FW2.2* in isogenic transgenic fruit are shown as fold change to corresponding WT fruit at 5 DBP and 2, 5, 10, and 20 DAP, respectively, in Figure 4. Insets in Figure 4 show the expression patterns for *SUN1*, *OVATE*, and *FW2.2* in WT floral buds and developing ovaries as fold-*ACTIN* (a house keeping gene) transcript levels. In WT floral tissues, the levels of *SUN1* and *OVATE* transcripts were upregulated at 5/20 DAP and 2 DAP, respectively, whereas *FW2.2* transcripts significantly increased at 10 and 20 DAP during floral and ovary development (Figure 4 inset). The patterns of *OVATE* and *SUN1* transcript levels in Ohio 8245 (WT) fruit is consistent with the WT fruit associated with the non-mutated *OVATE* gene [2]. Similar results were obtained in C15 fruits (Table S1).

A single base pair mutation from GAA to TAA in *OVATE* gene has been linked with functionality of this gene [2]. We checked the nucleotide sequence of *OVATE* in tomato cultivar Ohio 8245 and its isogenic transgenic lines C4, C15, and E8-8 to determine if WT or a mutated gene was residing in under-study genotypes. *OVATE* transcripts cloned from WT and three transgenic lines fruits showed absence of any mutation and thus would have more spherical shape phenotypes as observed in this investigation (Figure S1). Taken together, these results suggested that the observed obovoid fruit phenotype of transgenic line was associated with *ySpdSyn* transgene and not with the *OVATE* gene.

Patterns of transcript levels of the three fruit shape genes in transgenic lines (C4, C15 and E8-8) varied significantly compared to WT fruit (Figure 4; Table S1). *SUN1* transcripts were at higher levels in E8-8 than C4 or WT fruits at most stages of ovary development (Figure 4). The levels of *SUN1* transcripts in C4 were higher than WT at 2 DAP and 5 DAP stages but declined in 20 DAP ovaries in all the transgenic lines compared to WT fruit ovaries (Figure 4; Table S1). The *OVATE* transcript levels were significantly higher (>4-fold) in 5 DAP ovaries from C4 and E8-8 lines compared to WT ovaries. At other stages, the patterns of *OVATE* transcripts in transgenic ovaries were variable compared to the WT ovaries, with significantly higher levels at −5 and 10 DAP in C4 ovaries and at −5 and 20 DAP in E8-8 ovaries (Figure 4; Table S1). Consistent significant patterns for *FW2.2* transcripts levels in the fruits from C4 and E8-8 were not obtained, but generally, they were lower at all stages of ovary development (Figure 4, Table S1).

### 2.4. Expression Patterns of Selected Cell Division and Cell Expansion Genes in ySpdSyn Transgenic and Wild-Type Tomato

**Figure 5:**
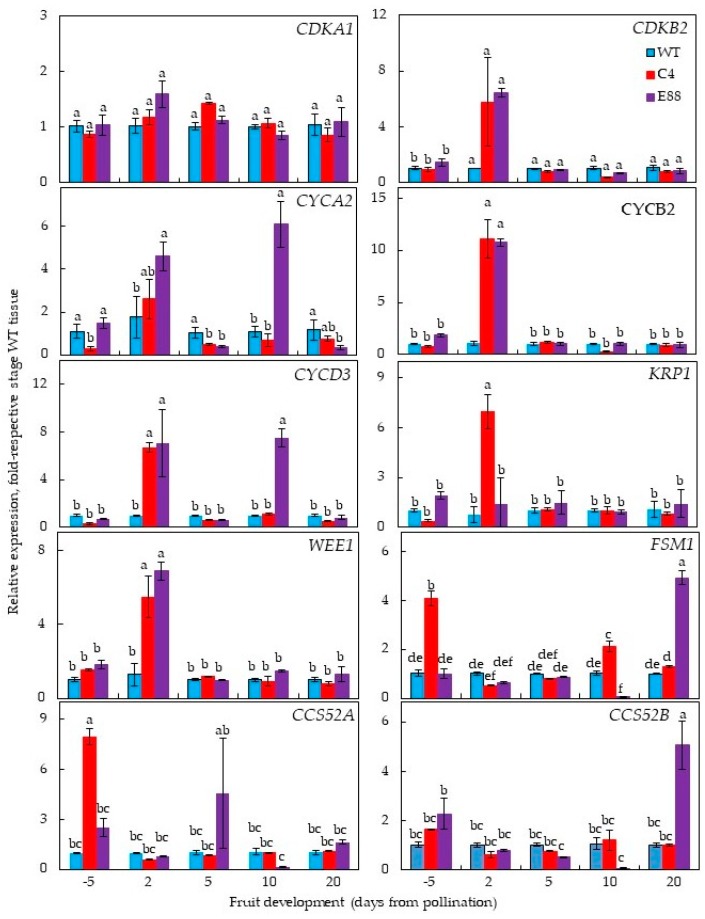
Changes in steady state transcript levels of cyclin-dependent kinases (*CDKA1* and *CDKB2*), cyclins (*CYCA2*, *CYCB2* and *CYCD3*), CDK1-inhibitors (*KRP1* and *WEE1*), and cell expansion-regulating genes (*FSM1*, *CCS52A* and *CCS52B*) in WT and *ySpdSyn*-expressing transgenic tomato floral buds and flower ovaries. Transcripts were quantified using qRT-PCR and relative expression levels were calculated by the 2^−ΔΔCт^ method using *SlACTIN* (Solyc04g011500.2.1) as housekeeping gene and plotted as fold-respective WT tissues. Other details were the same as described in the Figure 3 legend.

The relative steady state transcript levels of several genes implicated in regulating cell division and cell expansion were examined in floral buds and ovaries from WT, C4, and E8-8 lines. These included cyclin (*CYC*), cyclin-dependent kinases (*CDKs*), *FSM1* (inhibitor of cell expansion), and *CCS52A*/*CCS52B* (promoters of cell expansion) and their interacting partners *KPR1* and CDK inhibitor *WEE1*. In WT and transgenic flower and ovary tissues, the *CDKA1* transcript levels did not change significantly from 5 DBP to 20 DAP (Figure 5, Figure S2). In WT tissues, the *CDKB2* transcripts significantly decreased from 5 DBP to 2 DAP, followed by several-fold increase until 10 DAP before a precipitous decline at 20 DAP during WT fruit development (Figure S2). The two transgenic lines showed different pattern compared to WT tissues with the *CDKB2* transcripts being about 6-fold higher in C4 and E8-8 ovaries at 2 DAP compared to WT. Thereafter, a noticeable decrease in these genes occurred in all genotypes (Figure 5). In C15 tissues, the *CDKB2* transcripts significantly increased several-fold from 5 DBP to 2 DAP before precipitously declining at 20 DAP (Table S1).

Expression patterns for the three cyclins, *CYCA*2, *CYCB*2, *CYCD*3 (one from each of the three cyclin genes families), were different. Overall, their transcript levels were in the increasing order of *CYCB*2 > *CYCD*3 > *CYCA*2 (Figure 5, Figure S2). Levels of the *CYCA2* transcripts in WT ovaries were variable but not significantly different at any stage of floral and ovary development (Figure S2). In contrast, the transcript levels of *CYCB2* and *CYCD3* in WT tissues were about 4- and 12-fold higher, respectively, at 10 DAP before perceptible decline by 20 DAP (Figure S2). These transcript patterns in WT fruit are similar to that reported previously. The patterns of three cyclin gene transcripts in all transgenic ovaries were different. In the E8-8 tissues, several-fold increase in the *CYCA2* transcripts at 2 and 10 DAP stages was apparent, but this was not seen in C4 tissues (Figure 5). The steady state level of *CYCB2* was about 11-fold higher in both the transgenic lines compared to WT tissues at 2 DAP before declining notably in the transgenic tissues (Figure 5). The *CYCD3* transcripts levels had a bimodal pattern in E8-8 tissues showing about a 6-fold increase at 2 DAP and 5 DAP, while a 6-fold increase was obtained in C4 tissue only at 2 DAP (Figure 5). The steady state levels of all cyclin genes examined decreased considerably in 20 DAP tissues in all genotypes.

The pattern of *KRP1* (a CDK inhibitor) transcript accumulation did not change significantly during the floral and ovary development of WT (Figure S2). The C4 ovaries had 7-fold higher accumulation at 2 DAP, but in E8-8 ovaries, there was no change in *KRP1* transcripts at 2 DAP (Figure 5). The levels of CDK inhibitor gene, *WEE1*, increased until 10 DAP in WT (Figure S2), whereas in C4 and E8-8 tissues 6- to 7-fold increase in *WEE1* transcript levels was apparent at 2 DAP (Figure 5).

The transcript levels of *FSM1*, a cell expansion gene, increased several-fold in WT ovaries from 2 to 10 DAP and declined thereafter (Figure S2), a pattern similar to that reported in the facultative parthenocarpic line L-179 (*pat-2/pat-2*) [38]. The *FSM1* transcript levels in the C4 line were highest at 5 DBP (floral buds) and in E8-8 at 20 DAP compared to the other stages of development (Figure 5). The cell expansion promoting genes, *CCS52A* and *CCS52B*, had unique and differing expression patterns among the three genotypes analyzed (Figure 5). In WT tissues, transcript level of *CCS52A* was highest at 10 and 20 DAP and that of *CCS52B* was highest at 5 DBP and 10 DAP (Figure S2). The *CCS52A* transcript levels were significantly high at 5 DBP in C4 and E8-8, and at 5 DAP only in E8-8 ovaries (Figure 5). Highest levels of *CCS52B* transcripts during flower and ovary development was observed in E8-8 at 20 DAP (Figure 5).

### 2.5. Levels of Free and Bound PAs in Floral Buds and Developing Ovaries

**Figure 6:**
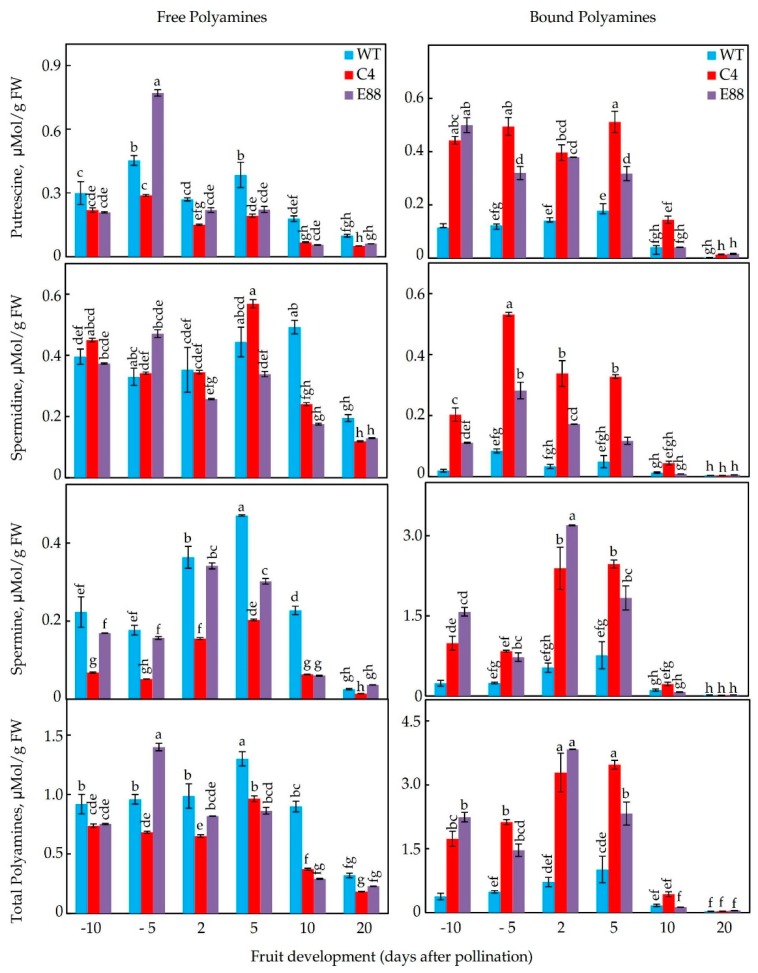
Changes in the levels of free and bound forms of putrescine, spermidine, and spermine (ɳmol/g FW) in floral buds and ovaries of WT and *ySpdSyn*-expressing transgenic tomato plants. Free and bound PAs were extracted and quantified by HPLC, as described in the Material and Methods section. Shown are average ± standard error (n ≥ 3 biological replicates). Similar letters above standard error bars indicate non-significant difference (at 95% confidence interval) among genotypes.

The free and bound levels of Put, Spd, and Spm were quantified in floral tissues of WT and the transgenic lines (C4, C15, E8-8) at −10, −5, 2, 5, 10, and 20 DAP (Figure 6, Table S2). Put, Spd, and Spm levels varied in all the three genotypes examined, with transgenic fruit having greater increase in bound forms of these PAs (Figure 6; Table S2). In the WT, the highest levels of free PAs were found at −5 and 5 DAP for Put, 5 and 10 DAP for Spd, and 5 DAP for Spm. The levels of free PAs declined dramatically at 20 DAP stage of WT fruit. In the transgenic lines, free Put levels were lower than the WT at all the developmental stages examined, except for the E8-8 fruit, which had significantly higher free Put level at 5 DBP than the other stages of development (Figure 6; Table S2). In contrast, levels of bound Put in transgenic lines increased several-fold from 10 DBP to 5 DAP and then decreased to levels comparable with WT at 20 DAP (Figure 6).

Free Spd levels in the WT line slighty increased from 10 DBP to 10 DAP stages and then declined at 20 DAP. For WT tissues, the content of bound Spd was several-fold lower than free Spd at all the fruit developmental stages (Figure 6). Compared to the WT, the free Spd levels had similar pattern of accumulation in developing floral buds and ovaries from the transgenic lines. The C4 and E8-8 fruit had the highest levels of free Spd at 5 DAP and 5 DBP, respectively (Figure 6). Remarkably, the bound Spd levels were high in all the transgenic flowers and developing ovaries at most stages of development from 10 DBP to 5 DAP, as compared to the WT line, but declined after 10 DAP in all the transgenic lines. The bound Spd levels peaked at 5 DBP in all three transgenic lines i.e., C4, C15, and E8-8 (Figure 6; Table S2).

The levels of free Spm in WT floral tissues were lower than its bound form throughout the development of floral and ovary tissues (Figure 6). Levels of free Spm increased from 5 DBP to 5 DAP before declining in both C4 and E8-8 genotypes (Figure 6). Like Spd, bound form of Spm in C4 and E8-8 was several-fold higher than the free form throughout the development of floral and ovary tissues. The pattern of bound form was similar in the three transgenic lines (Figure 6; Table S2). The highest bound Spm levels were found in E8-8 ovaries at 2 DAP (Figure 6). The C15 and C4 tissues had similar patterns (Table S2).

The levels of bound forms of Put, Spd, and Spm in WT tissues ranged from 2% to 52%, 4% to 88%, and 47% to 61% of that of total Put, Spd, and Spm, respectively. The percentage of bound-to-free PAs in the transgenic tissue had a wide range, varying from 41% to 293%, 5% to 233%, and 56% to 1630% for Put, Spd, and Spm, respectively (Figure 6). The 5 DBP and 2 DAP transgenic tissues contained up to 16-fold higher bound Spm compared to free Spm (Figure 6). These data indicate that expression of *ySpdSyn* under a fruit-specific promoter SlE8 (E8-8 line) or under a constitutive CaMV35S promoter (lines C4, C15) results in large accumulation of bound forms of Put, Spd, and Spm, as compared to the WT.

### 2.6. Transgene Increased Expression of PA Biosynthesis and Catabolic Pathway Genes

**Figure 7:**
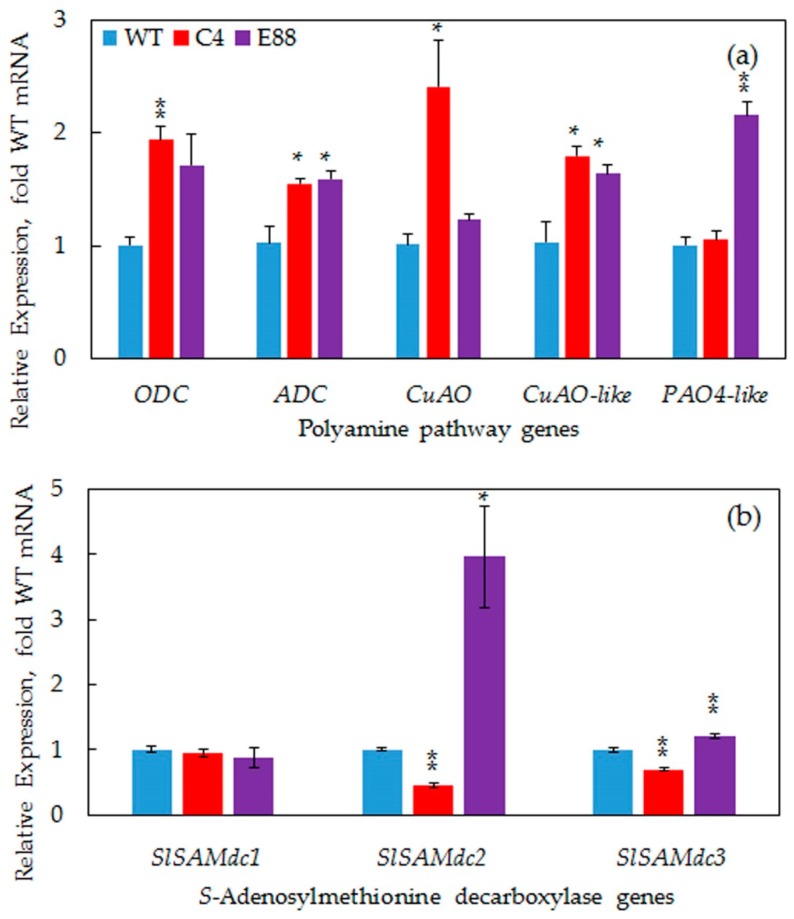
Steady state transcript levels of *ODC*, *ADC*, *Polyamine oxidases* (*CuAo*, *CuAo-like* and *PAO4-like*) and *SAMDC* genes (*SAMDC1*, *SMADC2*, *SAMDC3*) in WT and transgenic tomato ovaries 2 days after pollination. Transcripts were quantified using qRT-PCR and relative expression levels were calculated by the 2^−ΔΔCт^ method using *SlACTIN* (Solyc04g011500.2.1) as housekeeping gene and plotted as fold-respective to WT tissues. Other details were the same as in the Figure 3 legend.

We also quantified the levels of the biosynthesis and catabolic pathway genes of PAs in WT, C4 and E8-8 ovaries at 2 DAP. The steady state transcript levels of *ADC* and *ODC* genes increased significantly in 2 DAP ovaries in both C4 and E8-8 lines (Figure 7a). In 2 DAP ovaries, the transcripts of three tomato *SAMdc* genes were found to be differentially affected, with the steady state levels of *SAMDC2* and *SAMDC3* being higher in E8-8 line and slightly downregulated in the C4 line (Figure 7b). In contrast, the steady state levels of *SAMDC1* transcripts remained unchanged in response to transgene expression. Among the PA catabolic genes, significantly higher levels of *CuAO* and *CuAO-like* gene transcripts accumulated in the C4 line compared to the WT, whereas significantly higher levels of *CuAO-like* and *PAO4-like* gene transcripts were observed in 2 DAP ovaries from E8-8 lines (Figure 7a).

### 2.7. Statistical Analyses Accentuate Positive Correlations between Specific PAs levels, Gene Transcripts, and Fruit Architectural Parameters During Early Fruit Development

**Figure 8:**
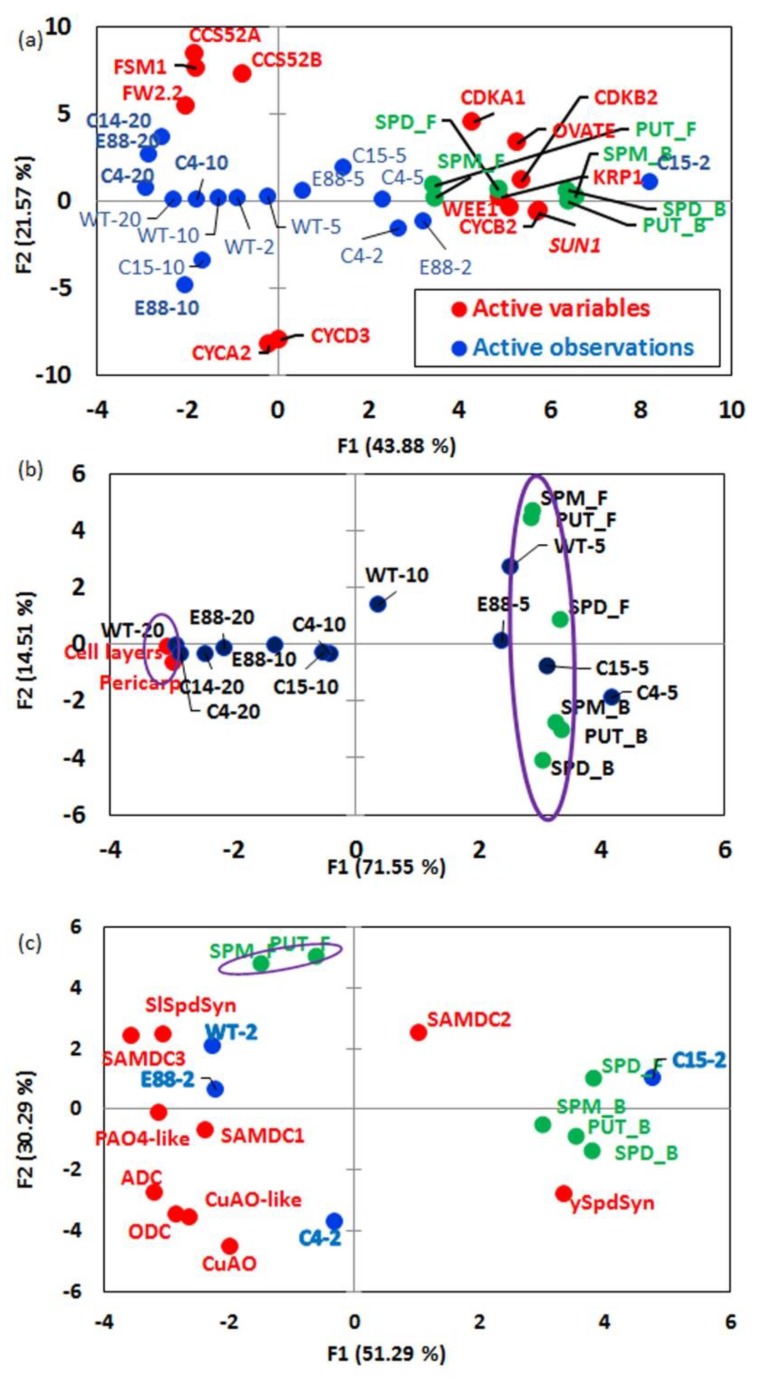
Principal component analyses (PCA) for polyamines (PAs), gene transcripts (cell division, cell expansion, fruit shape-realated and polyamine pathways) and cytological attributes of developing ovaries from WT and transgenic tomato lines. (**a**) PCA between free and bound forms of PAs and transcript levels of genes involved in cell division, cell expansion, and fruit shape development in tomato ovaries at 2, 5, 10 and 20 days after pollination (DAP); (**b**) PCA between free and bound forms of PAs and cell layers, and pericarp thickness of developing tomato ovaries at 5, 10, and 20 DAP; (**c**) PCA between free and bound forms of PAs and transcript levels of genes involved in PA biosynthesis (*ADC*, ODC, *SAMDCs* and *SlSpdSyn*/*ySpdSyn*) and catabolism (*CuAO*, *CuAO-like*, *PAO4-like*) in tomato ovaries at 2 DAP. Color codes: green—free and bound forms of PAs; blue—genotype and its ovary development stage (within parentheses); red—gene trasncripts or cells cytological attributes. Abbreviations: Put_F/B—free or bound form of Put; Spd_F/B—free or bound form of Spd; Spm_F/B—free or bound Spm.

Principal Component Analyses (PCA) of free and bound Put, Spd, and Spm content, together with expression levels of various cell division and cell expansion genes were measured at developmental stages from 2 DAP to 20 DAP (Figure 8). The free and bound forms of all three PAs clustered in the positive half of F1 along with *CYCB2*, *CDKA1*, *CDKB2* (two gene associated with cell division) and their inhibitors *KRP1* and *WEE1* clustered primarily with 2 and 5 DAP, the stages that regulate cell number in developing ovaries (Figure 8a). The observed clustering of cell expansion genes *CCS52A*, *CCS52B*, and *FSM1* predominately with 10 and 20 DAP stages and away from the free and bound forms of three PAs suggests that PA levels are inversely associated with fruit expansion phases (Figure 8a). Among the fruit shape genes, *SUN1* and *OVATE* expression were positively associated with free forms of all the three PAs quantified, whereas *FW2.2* was found to have a negative association (Figure 8a). The number of cell layers and pericarp thickness were associated with 10 and 20 DAP stages of the three transgenic lines, but not with WT fruit (Figure 8b). However, with further progression of ovaries from 10 DAP stage to 20 DAP stage, the number of cell layers and pericarp thickness were associated with both transgenic lines and WT (Figure 8b), suggesting that PAs cause early changes in these parameters.

During the cell division phase (2 DAP), free Spd and bound forms of the three PAs were found positively associated with *ySpdSyn* and *SAMDC2* transcripts and negatively with all other PA biosynthetic as well as catabolic gene transcripts examined (Figure 8c). The Pearson correlations coefficient analysis showed that free Spd levels were positively correlated (α ≥ 0.05) with transcript levels of *SUN1* and *OVATE*, while bound forms of Put, Spd and Spm were positively correlated with *SUN1*, *OVATE*, *CDKA1*, *CDKB2*, *CYCB2*, *KRP1*, and *WEE1* (Figure S3). Notably, transcripts of fruit shape gene *FW2.2* and cell cycle genes *CCS52A*, *CCS52B*, *FSM1*, *CYCA2*, and *CYCD3* did not have significant correlation with free or bound forms of PAs, suggesting a limited role of these cell cycle genes in affecting phenotype of fruit carrying FW2.2 mutation (Figure S4).

## 3. Discussion

We show here that PAs are cellular modifiers of fruit shape in tomato. This conclusion is based on the characterization of independent transgenic lines of tomato transformed with a heterologous *ySpdSyn* gene under two different promoters. The shape modification results in a more obovoid fruit shape, as assessed using three independent isogenic transgenic lines in comparison to the relatively spherical round shape of WT fruit (Figure 1a). The structural basis of altered morphology in the transgenic fruit is likely due to the reduction of cellular layers in pericarp, as the thickness of pericarp decreased by 50%, 54%, and 80% of WT in C15, E8-8, and C4 fruits, respectively (Figure 2c,d). Cytological examination of slices from ovaries at different stages of development revealed that PAs are associated with the periclinal cell division, leading to reduced number of cell layers in pericarp and the cell number in medial-lateral direction of pericarp, but not cell size per se. However, we could not conclude that reduced cellular layers and cell size are responsible for the obovoid shape and size of tomato fruit.

More extensive ultrastructure work should enable further understanding of the structural basis of diversity observed in tomato fruit. Altered fruit phenotypes have also been observed in mutants compromised in various growth and development processes, including flowering, fruiting, and photosynthesis, suggesting that many plant genes/proteins/processes influence fruit development [1,39]. Also, breeding for increased fruit size had previously led to large variations in fruit shape of cultivated tomato, from round to oblate, pear-shaped, torpedo-shaped, and bell pepper–shaped [1].

Several genes that affect tomato fruit shape have been identified and characterized [40]. Our results demonstrate that transgenic tomato with enhanced levels of PAs are altered in the expression of *SUN1* and *OVATE* during the early stages of development. Expression of *SUN1* leads to elongated fruit in tomato [17]. Expression of *SUN1* increased in the transgenic fruit, suggesting that it is associated with the elongation of fruit likely causing a more obovoid phenotype (Figure 4). In the mutated form, *OVATE* gene enhances obovoid over the round phenotype in tomato fruit [2]. By cloning *OVATE* mRNA from WT and three transgenic lines and determining DNA sequence of these clones, we have shown that the *OVATE* gene in Ohio 8245 tomato cultivar and its isogenic transgenic lines is a non-mutated type and would, therefore, favor a more spherical round fruit in Ohio 8245 cultivar (Figure S1). Thus, higher expression of this gene cannot be the cause of obovoid phenotype observed in the three transgenic lines. In addition to its phenotype, the *OVATE* gene is known to affect multiple factors in fruit, including metabolome, physiology, and fruit quality [41,42]. *Fw2.2* primarily affects fruit size as studies with transgenic fruit expressing WT *FW2.2* gene showed reduction in the size of fruit cv. Mogeor from a large one to a smaller one [6]. *FW2.2* has been also reported to have pleiotropic effects, especially on the distribution of photosynthate among all fruits on the plant [15]. The levels of *FW2.2* decreased significantly in transgenic fruit from 2 DAP to 20 DAP (Figure 4), which may result in the increase of fruit size and contribute to fruit shape. Expression of *SlSpdSyn* was upregulated during both the cell division and the cell expansion phases of fruit development, suggesting a dual role of Spd in fruit development (Figure 3).

It is generally accepted that the levels of free cellular PAs are under a tight genetic regulation to maintain PAs homeostasis during the growth and development of organisms [26,36,43]. Our data support this hypothesis. However, the role(s) of bound PAs in maintaining PAs homeostasis remains unknown. A nexus between PA levels, ratios of different PAs, and regulation of flower development is known [26]. Several mechanisms have been proposed to maintain the PAs homeostasis in organisms. These include biosynthesis, catabolism, conjugation, and transport of various PAs. Additionally, feedback inhibition and activation of PAs biosynthetic enzymes have been reported and suggested to play a role in establishing PA homeostasis. Due to the cationic nature of various PAs, their binding to various macromolecules in cellular milieu would also affect their cellular homeostasis [24]. Catabolism of PAs not only changes the levels of Put by back conversion of Spd and Spm, but also generates bioactive H_2_O_2_ [24]. Significant increases in the gene transcripts catalyzing PA catabolism, viz., *CuAO*, *CuAO-like*, and *PAO4-like* amine oxidases, suggest inter-conversion of Spm to Spd and Spd to Put (Figure 6), as reported by others [44,45]. PA depletion has also been implicated in a large number of plant responses and cytotoxic effects of excessive PAs [46,47]. Genetic regulation of PA homeostasis has been suggested as a means to maintain the cellular PAs concentration below toxic levels [43].

Expression of *SlSpdSyn* was upregulated both during the cell division and the cell expansion phase of fruit development, suggesting a dual role of PAs in fruit development (Figure 3). A large increase in bound forms of Put, Spd, and Spm in transgenic ovaries was observed in this study. However, the chemical nature of the bound PAs still needs to be investigated (Figure 6). The bound forms of various PAs could represent formation of conjugates or increased ionic binding to protein, RNA, DNA, and other chemical moieties. The molecular basis of enhanced Put, Spm, and Spm in such bound forms is not known, but would likely result from enhanced biosynthesis of PAs, especially when PA biosynthesis genes are upregulated, as observed in this investigation (Figure 7). However, the role of catabolism in this scenario remains to be determined. Elucidation of the chemical basis of massive accumulation of bound PAs in transgenic fruits should help understand their role in PAs homeostasis. In this context, it is known that PAs induce Spd/Spm *N1*-acetyltransferase and efflux transporters and likely self-regulate their levels [48,49,50,51].

## 4. Materials and Methods

### 4.1. Plant Material and Growth Conditions

Wild type and transgenic tomato lines homozygous for *ySpdSyn* gene fused to either constitutive CaMV35S promoter (lines C4 and C15) or fruit/ethylene-specific SlE8 promoter (line E8-8) have been described previously [27]. These plants were grown in high porosity potting mix (52Mix, Conrad Fafard, Inc., Agawam, MA, USA) in a greenhouse with 16 h day/8 h night photoperiod and 23 °C day/18 °C night temperature. Tomato flowers and fruit developmental stages were tagged. Samples were collected on day 10 and day 5 before pollination (DBP) and 2, 5, 10, and 20 days after pollination (DAP) (Figure S4), immediately frozen in liquid N_2_ and stored at −80 °C until further use. To determine fruit shape index, fruits at various stages of development were sliced from proximal-distal axis, scanned, and analyzed using ‘Tomato Analyzer’ software [52].

### 4.2. Cytological Analysis

Axis slices of fresh tissues were fixed in 10% formalin (pH 6.8–7.2) and processed in Tissue-Tek VIP^®^ (Sakura Finetek USA Inc., Torrance, CA, USA) using following sequential treatments: 70% ethanol for 50 s, 95% ethanol for 50 s (2 cycles), 100% ethanol for 33 s (3 cycles), toluene for 60 s (2 cycles), and paraffin for 45 s at 63 °C (4 cycles). For deparaffinization, tissues were dipped in xylene for 5 min (2 cycles), 100% ethanol for 2 min, 95% ethanol for 2 min, and 70% ethanol for 2 min, followed by rehydration in deionized water. Tissues were embedded in paraffin using Cryo-therm (Lipshaw, Tucker, GA, USA) and 5–7 µm thick sections were made using microtome (Finesse ME, Thermo Electron, Waltham, MA, USA). These were then stained with 1% toluidine blue–O, sectioned, dehydrated by serial quick dips in 70%, 95%, and 100% ethanol, and xylene [53]. Slides were scanned with ScanScope CS (Aperio Technologies Inc., Buffalo Grove, IL, USA) at different magnifications, 40x being the maximum. The digital images were analyzed for cell number and cell layers using ImageScope 11 (Aperio Technologies Inc., Buffalo Grove, IL, USA), and for cell size by ImageJ [54]. Three independent biological replicates were analyzed at each stage from each genotype.

### 4.3. Transcript Analysis by Quantitative Real-Time PCR

For qRT-PCR, the frozen tissues were ground to powder using liquid N_2_, total RNA was extracted from 100 mg tissue powder using QIAzol^®^ Lysis reagent (Qiagen Sciences, Germantown, MD, USA) and purified using RNeasy^®^ Mini Kit (Qiagen Sciences, Germantown, MD, USA). The RNA samples were treated with one unit RQ1 RNase-free DNase (Promega Corporation, Madison, WI, USA) and first-strand cDNA was synthesized using SuperScript II Reverse Transcriptase (Invitrogen, Waltham, MA, USA). GoTaq^®^ qPCR Master Mix (Promega Corporation, Madison, WI, USA) was used in qRT-PCR reaction mixture. All primers and cDNA templates were optimized for gene expression analysis according to the 2^−ΔΔCT^ method [55]. StepOnePlus^TM^ Real-Time PCR System (Applied Biosystems, Waltham, MA, USA) was used with program sequence as follows: 95°C for 10 min; 95 °C for 15 s and 60 °C for 60 sec (40 cycles); 95 °C for 15 s; and 60 °C for 60 s. Comparative C_T_ values of gene expression were quantified using StepOne^TM^ 2.0 software (Applied Biosystems, Waltham, MA, USA). *ACTIN* was used as standard housekeeping gene to normalize the expression of target genes. Accession numbers of all genes with their primer sequences used in this study are listed in Table S3. All data presented here represent average ± standard error of a minimum of three independent biological replicates.

### 4.4. Quantification of Polyamines by High Pressure Liquid Chromatography

Polyamines were extracted from floral buds and fruit tissues of tomato plants and dansylated as described previously [56] with some modifications. Briefly, 200 mg of finely ground sample was homogenized in 800 µL of 5% ice-cold perchloric acid (PCA) using a hand-held homogenizer precooled at 4 °C for 1 h. The homogenate was centrifuged at 20,000 *g* for 30 min at 4 °C and both the supernatant and residue were collected separately. PAs present in the supernatant were labeled as free PAs. To quantify PCA-insoluble bound PAs, the 20,000 *g* pellet was washed twice with 5% PCA, re-suspended in 800 µL of 5% cold PCA, and then hydrolyzed in an equal volume of 6N HCl for 18 h at 110 °C. Saturated sodium carbonate (200 µL) and 1,7-heptanediamine (400 µL, as an internal standard) were added to the 100 µL supernatant or the hydrolyzates, and then dansylated with dansyl chloride for 60 min at 60 °C in the dark. Dansylation was terminated by adding 100 µL proline and incubating the reaction mixture for 30 min at 60 °C. Dansylated PAs were extracted in 500 µL toluene, air dried, and dissolved in 250 µL acetonitrile. The samples were then diluted four times with acetonitrile, filtered through a 0.45 µm syringe filter (National Scientific, Claremont, CA, USA), and fractionated on a reversed-phase Nova-Pak C18 column (3.9 × 150 mm, 4.0 µm pore size) using Waters 2695 Separation Module equipped with Waters 2475 Multi λ fluorescence detector (excitation 340 nm, emission 510 nm) with a binary gradient composed of solvent A (100% Water) and solvent B (100% acetonitrile) at 1 mL/min. Initial conditions were set at 60:40 (A:B) and then a linear gradient was processed with conditions set at 30:70 (A:B) at 3 min; 0:100 (A:B) at 10 min, and 60:40 (A:B) at 12 min. The column was flushed with 60:40 (A:B) for 3 min before the next sample injection. To determine PAs recovery and generate calibration curves, authentic PA standards (Sigma-Aldrich, St. Louis, MO, USA) were used as control. PAs were integrated and quantified using Millennium^32^ 4.0 from Waters Corporation. After hydrolysis, PCA-soluble and PCA-insoluble samples were quantified and are designated as free and bound forms of each PA, respectively, throughout the manuscript.

## 5. Conclusions

**Figure 9:**
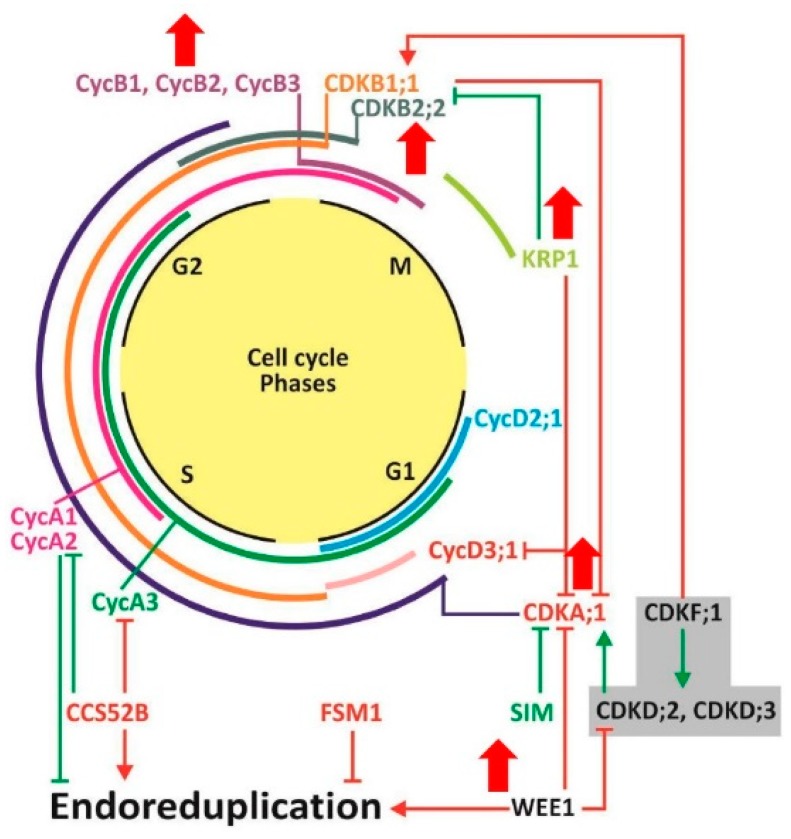
A model showing the polyamine-mediated changes in transcript levels of genes involved in cell cycle progression and endoreduplication during tomato fruit development. In eukaryotes, cell cycle is mainly comprised of interphase and mitosis (M phase). Interphase is further divided into three phases: i) G1 (Gap 1) during which cell increases in size and becomes ready for DNA synthesis; ii) S (Synthesis) where DNA replication occurs; and iii) G2 (Gap 2) during which cell either continues to grow until ready for mitosis or enter into DNA amplification phase, called endoreduplication [58,59]. Expression levels of cyclins or CDKs during different phases of cell cycle progress are indicated in colors. CDK-activating kinases are highlighted in gray box. Transcript levels of genes enhanced by higher polyamines (Spd/Spm) are indicated with upward-facing thick red arrows. Abbreviations: G—Gap phase; M—Mitosis phase; S—Synthesis phase; Cyc—Cyclins; CDKs—Cyclin dependent kinases; KRPs—Kip-related proteins.

Our findings on gene transcripts regulating cell division and cell enlargement in response to the expression of *ySpdSyn* transgene and subsequent alteration in PAs levels are summarized in a model (Figure 9). The cellular levels of bound Put, Spd, and Spm correlate with *CDKA1*, *CDKB2*, *CYCB2*, *KRP1*, and *WEE1* transcripts, but not *CCS52A*, *CCS52B*, *CYCA2*, and *CYCD3* genes in transgene-associated change in fruit shape. The patterns of gene expression during early fruit development indicate that the upregulated *CDKB2* may in turn activate *CYCB2* expression during ovary development at 2 DAP and 5 DAP stages, the phase of active cell division of pollinated ovaries. The precipitous decrease in the expression of all cyclins in 20 DAP tomato fruit is consistent with the end of cell division phase at this stage of fruit development [37]. It is noted here that fruit shape changes occur at an early stage of fruit development (before 5 DAP), during enhanced accumulation of PAs, and continue to persist during fruit development and ripening, as was also found to be the case for *SUN1* and *OVATE* transgenic fruits. Our results indicate that the expression of the transgene is associated with reduced cell division in the medial-lateral direction of fruit, which can cause more elongated fruit shape, an effect also seen in tomato fruit over-expresser for *SUN1* gene [17,57]. In our studies, the parental Ohio 8245 tomato cultivar expressed a functional *OVATE* gene, which would reduce cell division at the distal end and induce more round fruit shape. Thus, increase in obovoid shape of the transgenic fruit seems to be linked to both *SUN1* and *OVATE* genes, with *SUN1* likely having a stronger effect on the fruit phenotype.

## Supplementary Materials

The following are available online at https://www.mdpi.com/2223-7747/8/10/387/s1, Figure S1: Sequence alignment of *OVATE* gene in WT and transgenic fruits with its mutated version (*ovate*) containing stop codon (TAA); Figure S2: Expression patterns of various cell division and cell expansion genes in WT developing floral and ovary tomato tissues; Figure S3: Correlation coefficient analysis of PA levels and transcripts of genes involved in fruit shape, cell cycle progression, cell expansion and polyamine biosynthesis and catabolism; Figure S4: Representative flower and fruit developmental stages registered in *Solanum lycopersicum* cv. Ohio8245; Table S1: Steady state transcript levels of cell division, cell expansion, fruit shape and polyamine pathway genes at various developmental stages of floral and ovaries tissues in transgenic tomato line C15; Table S2: Free and bound forms of putrescine, spermidine and spermine in floral buds and ovaries tissues at various developmental from wild-type (WT) and *35S:ySpdSyn* expressing transgenic tomato line C15; Table S3: List of genes and their primer sequences used for quantitative real-time PCR analyses. References [60] are cited in the Supplementary Materials.
