# Supplementary material for: Fruit Architecture in Polyamine-Rich Tomato Germplasm Is Determined via a Medley of Cell Cycle, Cell Expansion, and Fruit Shape Genes
Source: Plants (Basel). 2019 Sep 29;8(10):387. doi: 10.3390/plants8100387 (PMC6843802; doi:10.3390/plants8100387)
Supplement: Supplementary file 1 [file plants-08-00387-s001.pdf]

```
WT                           GGGAAAGTGAAGGAGAGTTTCGCGATAGTGAAGAAATCTCAGGACCCGTACGAAGATTTCA
C4                           GGGAAAGTGAAGGAGAGTTTCGCGATAGTGAAGAAATCTCAGGACCCGTACGAAGATTTCA
C15                          GGGAAAGTGAAGGAGAGTTTCGCGATAGTGAAGAAATCTCAGGACCCGTACGAAGATTTCA
E88                          GGGAAAGTGAAGGAGAGTTTCGCGATAGTGAAGAAATCTCAGGACCCGTACGAAGATTTCA
Solyc02g085500.2.1           GGGAAAGTGAAGGAGAGTTTCGCGATAGTGAAGAAATCTCAGGACCCGTACTAAGATTTCA
                             *************************************************** *********

WT                           AGAGATCGATGATGGAAATGATTTTAGAGAAGGAAATGTTTGAGAAGAATGAGCTGGAACA
C4                           AGAGATCGATGATGGAAATGATTTTAGAGAAGGAAATGTTTGAGAAGAATGAGCTGGAACA
C15                          AGAGATCGATGATGGAAATGATTTTAGAGAAGGAAATGTTTGAGAAGAATGAGCTGGAACA
E88                          AGAGATCGATGATGGAAATGATTTTAGAGAAGGAAATGTTTGAGAAGAATGAGCTGGAACA
Solyc02g085500.2.1           AGAGATCGATGATGGAAATGATTTTAGAGAAGGAAATGTTTGAGAAGAATGAGCTGGAACA
                             *************************************************************

WT                           GCTTTTACAATGTTTTCTGTCGTTGAACGGAAAGCATTATCATGGAGTGATAGTTGAGGCG
C4                           GCTTTTACAATGTTTTCTGTCGTTGAACGGAAAGCATTATCATGGAGTGATAGTTGAGGCG
C15                          GCTTTTACAATGTTTTCTGTCGTTGAACGGAAAGCATTATCATGGAGTGATAGTTGAGGCG
E88                          GCTTTTACAATGTTTTCTGTCGTTGAACGGAAAGCATTATCATGGAGTGATAGTTGAGGCG
Solyc02g085500.2.1           GCTTTTACAATGTTTTCTGTCGTTGAACGGAAAGCATTATCATGGAGTGATAGTTGAGGCG
                             *************************************************************

WT                           TTCTCAGACATTTGGGAGACTTTGTTTTTAGGTAATAATGATAGAGTAAGGAGGATGTCAA
C4                           TTCTCAGACATTTGGGAGACTTTGTTTTTAGGTAATAATGATAGAGTAAGGAGGATGTCAA
C15                          TTCTCAGACATTTGGGAGACTTTGTTTTTAGGTAATAATGATAGAGTAAGGAGGATGTCAA
E88                          TTCTCAGACATTTGGGAGACTTTGTTTTTAGGTAATAATGATAGAGTAAGGAGGATGTCAA
Solyc02g085500.2.1           TTCTCAGACATTTGGGAGACTTTGTTTTTAGGTAATAATGATAGAGTAAGGAGGATGTCAA
                             *************************************************************

WT                           TTCATGAT
C4                           TTCATGAT
C15                          TTCATGAT
E88                          TTCATGAT
Solyc02g085500.2.1           TTCATGAT
                             ********
```

Figure S1: Sequence alignment of *OVATE* gene in WT and transgenic fruits with its mutated version (*ovate*) containing stop codon (TAA). Multiple sequence alignment was generated using Clustal Omega (1.2.0). Three base pair mutation (T/GAA) in sequence in indicated in red box.

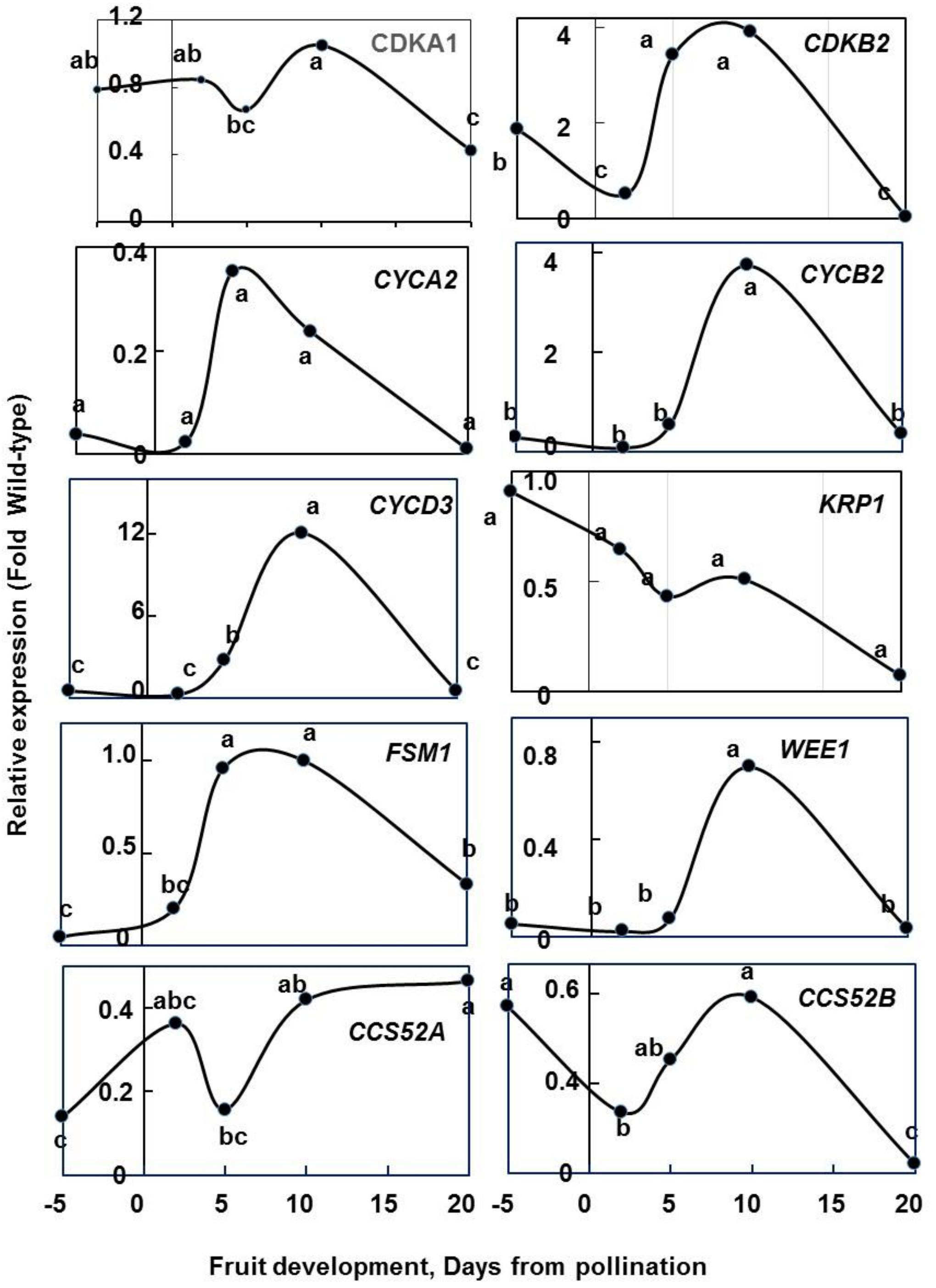

Figure S2: Expression patterns of various cell division and cell expansion genes in WT developing floral and ovary tomato tissues.

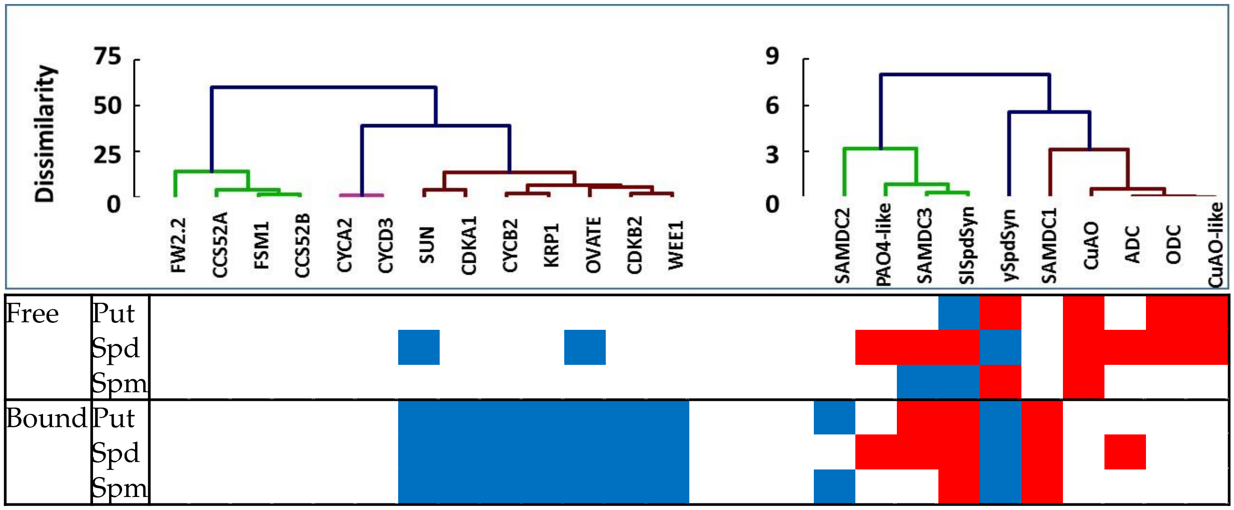

Figure S3: Correlation coefficient analysis of PA levels and transcripts of genes involved in fruit shape, cell cycle progression, cell expansion and polyamine biosynthesis and catabolism. Changes in gene expression and levels of different forms of various PAs were analyzed using XLSTAT version 2014.4.06 to determine the correlation and dendrogram (arrangement of the clusters among genes).

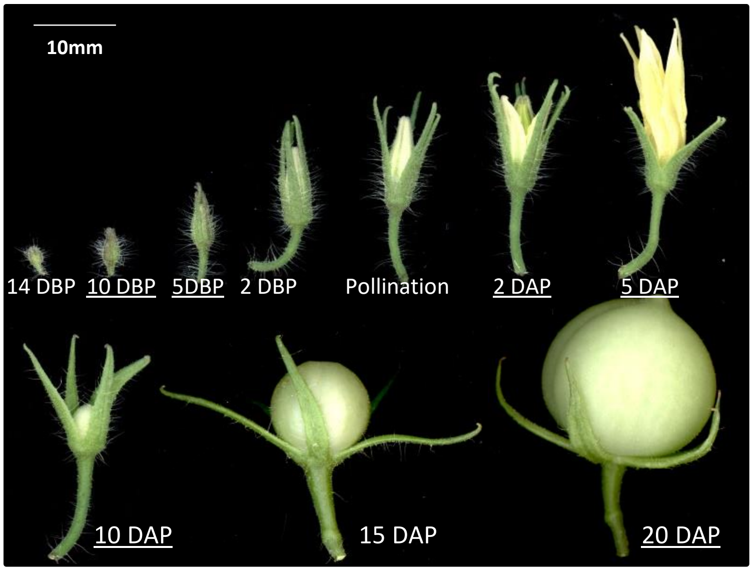

Figure S4: Representative flower and fruit developmental stages registered in *Solanum* l*ycopersicum* cv. Ohio8245. Underlined flower and fruit samples at 10 and 5 days before pollination (DBP) and 2, 5, 10 and 20 days after pollination (DAP) were used in cytological, transcriptional and polyamine analyses.

Table S1: Steady state transcript levels of cell division, cell expansion, fruit shape and polyamine pathway genes at various developmental stages of floral and ovaries tissues in transgenic tomato line C15. Transcripts were quantified using qRT-PCR and relative expression levels were calculated by the $2^{-\Delta\Delta C_T}$ method using *SlACTIN* (Solyc04g011500.2.1) as housekeeping gene and plotted as fold-respective WT tissues.

	**Tomato fruit development stages**				
**Genes**	**5 DBP**	**2 DAP**	**5 DAP**	**10 DAP**	**20 DAP**
ySpdSyn	188.5	61.6	4971.4	135.7	116.6
SlSpdSyn	0.9	0.4	1.4	5.8	1.6
OVATE	1.3	9.6	4.4	0.5	2.7
SUN	0.7	6.7	5.2	2.3	0.8
FW2.2	2.3	0.4	1.7	0.0	0.8
CDKA1	0.9	1.5	1.8	0.6	1.3
CDKB2	0.9	9.2	1.0	0.3	1.7
CYCA2	1.7	0.9	0.6	4.0	0.9
CYCB2	0.9	15.3	2.0	0.8	0.9
CYCD3	0.7	1.7	1.1	3.1	0.4
KRP1	1.0	6.7	1.6	0.7	1.6
WEE1	1.1		0.9	8.9	1.3
FSM1	1.1	1.5	2.8	0.3	6.0
CCS52A	1.3	0.8	0.9	0.2	2.2
CCS52B	1.2	2.3	1.2	0.0	4.3
ODC		0.28			
ADC		0.36			
CuAO		0.35			
CuAO-like		0.58			
PAO4-like		0.30			
SAMDC1		0.82			
SAMDC2		3.15			
SAMDC3		0.67			

Abbreviations: DBP – days before pollination; DAP – days after pollination.

Table S2: Free and bound forms of putrescine, spermidine and spermine in floral buds and ovaries tissues at various developmental stages from wild-type (WT) and *35S:ySpdSyn* expressing transgenic tomato line C15.

**Stage**	**5 DBP**		**2 DAP**		**5 DAP**		**10 DAP**		**20 DAP**	
**Transgenic line**	**WT**	**C15**	**WT**	**C15**	**WT**	**C15**	**WT**	**C15**	**WT**	**C15**
Free putrescine	283	453	223	270	207	385	62	179	39	99
Free spermidine	399	330	597	353	482	444	227	492	113	195
Free spermine	67	177	267	363	201	471	53	227	15	25
Bound putrescine	460	123	653	141	324	178	123	41	13	2
Bound spermidine	571	127	876	51	362	74	41	20	5	7
Bound spermine	1055	238	4463	526	1686	758	127	108	13	18
Free total polyamines	749	959	1087	986	890	1299	342	898	167	319
Conjugated total polyamines	142	52	522	312	359	703	138	0	10	0
Bound total polyamines	2086	487	5991	718	2372	1009	291	168	31	27
Total polyamines	2978	1498	7601	2016	3621	2379	770	1386	208	1037

Table S3: List of genes and their primer sequences used for quantitative real-time PCR analyses.

**Gene ID**	**Abbreviated Name**	**Forward Primer (5' to 3')**<br>**Reverse Primer (5' to 3')**
Solyc02g085500.2.1	*OVATE* [2]	GAGCTACCGGCAAGGTTATCG<br>CACTATCGCGAAACTCTCCTTCA
Solyc10g079240.1.1	*SUN1*	CAAACAGCACAGCGAAGCAA<br>TGGCGCTGTCATACATTTCAC
Solyc02g090730.2.1	*FW2.2*	TTTGCTGGGATTGACAGGATT<br>CAAGGTGCCTCTTCCAGATCA
Solyc08g066330.1.1	*CDKA1*	ACTGCTTGGATCACGCCATT<br>AACAGAGGCGGCTGATTCAC
Solyc04g082840.2.1	*CDKB2*	AGTGACAAACCAAGCCCTCTTC<br>CCCAGGCCAGAGTTCTTCATT
Solyc06g065680.2.1	*CYCA2*	CCAAAAGACCAGCCCCAGAT<br>CGCTTAGGCTGTTGAGAAGCA
Solyc02g082820.2.1	*CYCB2*	AAGGCAGCAACAGGGAAACTAA<br>GGCTCACACTTGGCTGCATA
Solyc02g092980.2.1	*CYCD3*	AACATGATGAGCTTGCCACACT<br>CCCCCATTAAAGACCCATCTG
Solyc09g091780.2.1	*KRP1*	GGAGAGCACACCTTGCAGTTT<br>TACTCTGCCGTTGGCCTCAT
Solyc09g074830.2.1	*WEE1*	GCCTCTTCTTCCGGGTCACT<br>TGCAGAAGGACGACGTGTTG
Solyc05g005710.2.1	*SlSpdSyn*	GGAGGAGGAGATGGTGGTGTCC<br>GCAACTCCGTCACCAATGTGGAGAT
Solyc10g052470.1.1	*FSM1*	GGGATGTTTTCTTTATTGACAATGG<br>CAGAGGTGGAATTATGGGATCCT
Solyc08g080080.2.1	*CCS52A* [60]	CTCTGACAGGTCATACATATAGA<br>ACAATTGTCTGTCCATCTGGAG
Solyc06g043150.2.1	*CCS52B* [60]	TCCTGCAGCAGTGAAGGAC<br>TCCTGCGTCTTCCTTGATTT
Solyc04g082030.1.1	*ODC*	TGCGAGCTTTTGCTTCGAAT<br>GGTAATGCGCCGTATTTTGG
Solyc10g054440.1.1	*ADC*	CTCGGCGGACTCCATAACC<br>GCCCAGGGACTGCATAGGT
Solyc08g079430.2.1	*CuAO*	CGATTTCCCCAATCATCCTTT<br>CCGCAATTGAATGAACGATTT
Solyc05g013440.2.1	*CuAO-like*	CAATCGCACTGGGCAGTTAA<br>CTCCTCAAGAATTTTGCCTCTGA
Solyc02g081390.2.1	*PAO4-like*	CCACTTCATATGCTTGCGGTTA<br>TCGAGGTCACAAGCAAGTCTTC
YPR069C	*ySpdSyn*	AGCCACCGAAAGGGATGAATTTGC<br>ACATAACCAGGCTTCCTCAACGGA
Solyc04g011500.2.1	*Actin*	TGG TCG TAC CAC CGGTAT TGTG<br>AATGGCATGTGGAAGGGCATA C
